# A scan statistic to extract causal gene clusters from case-control genome-wide rare CNV data

**DOI:** 10.1186/1471-2105-12-205

**Published:** 2011-05-26

**Authors:** Takeshi Nishiyama, Kunihiko Takahashi, Toshiro Tango, Dalila Pinto, Stephen W Scherer, Satoshi Takami, Hirohisa Kishino

**Affiliations:** 1 Doctor of Public Health Program in Biostatistics, National Institute of Public Health, Wako, Saitama 351-0197, Japan; 2 Clinical Trial Management Center, Nagoya City University Hospital, Nagoya 467-8601, Japan; 3 Department of Technology Assessment and Biostatistics, National Institute of Public Health, Wako, Saitama 351-0197, Japan; 4 Center for Medical Statistics, Tokyo 105-0021, Japan; 5 The Center for Applied Genomics and Program in Genetics and Genomics Biology, Hospital for Sick Children, Toronto, Ontario M5G 1L7, Canada; 6 Laboratory of Biometry and Bioinformatics, Graduate School of Agriculture and Life Sciences, University of Tokyo, Tokyo 113-8657, Japan

## Abstract

**Background:**

Several statistical tests have been developed for analyzing genome-wide association data by incorporating gene pathway information in terms of gene sets. Using these methods, hundreds of gene sets are typically tested, and the tested gene sets often overlap. This overlapping greatly increases the probability of generating false positives, and the results obtained are difficult to interpret, particularly when many gene sets show statistical significance.

**Results:**

We propose a flexible statistical framework to circumvent these problems. Inspired by spatial scan statistics for detecting clustering of disease occurrence in the field of epidemiology, we developed a scan statistic to extract disease-associated gene clusters from a whole gene pathway. Extracting one or a few significant gene clusters from a global pathway limits the overall false positive probability, which results in increased statistical power, and facilitates the interpretation of test results. In the present study, we applied our method to genome-wide association data for rare copy-number variations, which have been strongly implicated in common diseases. Application of our method to a simulated dataset demonstrated the high accuracy of this method in detecting disease-associated gene clusters in a whole gene pathway.

**Conclusions:**

The scan statistic approach proposed here shows a high level of accuracy in detecting gene clusters in a whole gene pathway. This study has provided a sound statistical framework for analyzing genome-wide rare CNV data by incorporating topological information on the gene pathway.

## Background

In recent years, it has become evident that structural genetic variants, most of which appear to be in the form of copy-number variants [also called copy-number variations (CNVs)], can cause various common diseases, most notably neurodevelopmental diseases [1-12]. Although common CNVs typable on current commercially available platforms are unlikely to play major roles in the pathogenesis of common diseases [13], rare CNVs are suggested to be involved in susceptibility to common diseases, either individually or collectively [14].

Recently, rare variants, including rare CNVs, have received much attention in the context of "missing heritability," which indicates that common variants identified to date typically explain only a small fraction of the overall heritability [15]. However, statistical methods to analyze rare variants, including rare CNVs, are under development [16-22]. In this study, we report a statistical method for case-control data of genome-wide rare CNVs.

Because any given CNV has an extremely low population frequency, the statistical power to detect an individual rare CNV associated with a disease is limited. This fact motivates analytical approaches that test the combined effect of multiple rare CNVs. The simplest version of such analyses compares the frequencies of rare CNVs per individual or the frequencies of genes affected by rare CNVs per individual between cases and controls [4,5,10,12]. This analysis, termed CNV burden analysis, does not identify the genes that cause an apparent association. Therefore, to identify causal genes from multiple rare CNVs, methods termed gene set analyses that compare the proportion of genes affected by rare CNVs in an *a priori *defined gene set (*e.g.*, a gene set in the same biological pathway) between cases and controls, are used [5,8,10,12]. In these methods, typically, hundreds of gene sets are tested, and the tested gene sets are often found to overlap (*i.e.*, the same genes in multiple gene sets). Consequently, the statistical power of such tests decreases after adjustment for multiple testing, and the results obtained are difficult to interpret, particularly when many gene sets show statistical significance.

Gene set analyses do not use the topological features of functional gene pathways, such as those defined by the Kyoto Encyclopedia of Genes and Genomes (KEGG) [23] or MSigDB [24]. Genes causing any one disease can be reasonably assumed to be functionally related and therefore closer to each other in a pathway compared to noncausal genes [25].

Based on this highly probable assumption, we propose a cluster detection test to detect disease-associated gene sub-pathways from a whole pathway using case-control rare CNV data. For this cluster detection test, we used a scan statistic framework, first presented by Naus [26], which has been applied in many different fields including epidemiological studies for detecting disease clustering [27-29]. To apply a scan statistic framework to the context of rare CNVs, we employed methods for association testing with rare nucleotide variants [16-18,20]. Use of the proposed method has been illustrated with a real dataset in which our approach completely avoids multiple testing and the difficulties in interpreting overlapping gene sets derived from typical gene set analyses. We also evaluated the performance of the proposed methodology in a simulation study, which assumes that any one gene disrupted by CNV in a causal gene sub-pathway (a gene cluster) may lead to a common disease.

## Results

### Application to an empirical dataset

We applied the proposed test to a published case-control rare CNV study of autism spectrum disorder (ASD) [12]. The original paper details the dataset information. In brief, genome-wide scans for CNVs greater than 30 kb were performed on genomic DNA from 1275 ASD cases and 1981 controls using Illumina Infinium 1 M single arrays. Considering rare CNVs that were present in less than 1% of the sample, 5478 CNVs present in 996 cases and 1287 controls of European ancestry were included in this study after stringent quality-control criteria were consistently applied between cases and controls.

Using the gene pathway defined by Pathway Commons [30], we could identify a statistically significant gene cluster only for deletions (*p-valu*e = 0.025) but not for all CNVs (*p-valu*e = 0.055). Note that a deletion was defined as the loss of one copy (heterozygous deletion) or two copies (homozygous deletion). Because only deletions were significantly enriched in gene sets in cases over controls in the original paper [12], we first analyzed all CNVs and, subsequently, analyzed the copy-number losses (deletions) in this analysis. The detected gene cluster includes 776 genes within a radius of two, which is centered on the ACAT1 gene (Additional file 1, Additional file 2).

**Figure 1:**
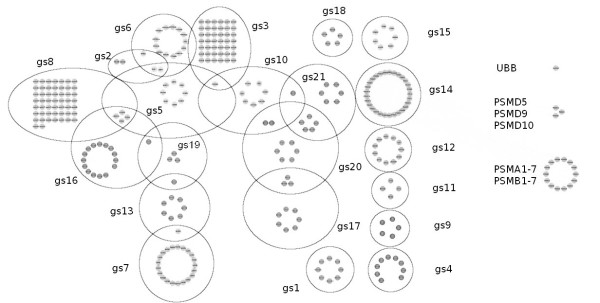
**Mutually Overlapping Gene Sets Listed in Table 1**. Colored nodes represent genes in a gene set, and the name of the gene set (gs1-21) represents the ranking in Table 1. Fourteen genes (PSMA1-7/PSMB1-7), which are shown on the right side of the figure, are shared by 11 gene sets (gs1, gs2, gs3, gs5, gs6, gs8, gs9, gs12, gs14, gs15, and gs16). Similarly, three genes (PSMD5, PSMD9, and PSMD10) are shared by nine gene sets (gs2, gs3, gs5, gs6, gs8, gs9, gs12, gs14, and gs16), and the remaining gene (UBB) is shared by eight gene sets (gs2, gs3, gs5, gs6, gs8, gs9, gs12, and gs16).

To interpret this gene cluster, we examined the overlapping proportion between this gene cluster and predefined gene sets. For this purpose, we used all 3272 curated gene sets in the Broad Institute's MSigDB ver 3.0 [24]. Table 1 and Figure 1 list the gene sets overlapping with the detected gene cluster at a proportion of more than 50%. In Table 1, three gene sets (the 4th, 13th, and 19th gene sets from the top) are specific to a particular disease, *e.g.*, malignant glioma or liver cancer and are not useful for interpreting the biological function of the detected gene cluster. Eight of the 18 remaining gene sets (the 1st, 2nd, 3rd, 6th, 8th, 9th, 15th, and 18th gene sets from the top) are ubiquitin-related. The relationship between ubiquitin and ASD has been postulated and recognized [31,32]. The ubiquitin-proteasome system operates in pre- and postsynaptic compartments and regulates synaptic attributes, including neurotransmitter release, synaptic vesicle recycling in presynaptic terminals, and dynamic changes in dendritic spines and in postsynaptic density [33]. We noted that UBE3A, PARK2, RFWD2, and FBXO40 of the ubiquitin gene family were significantly enriched in ASD cases according to case-control CNV analyses [9]. These results suggest that our approach correctly extracted a potential disease susceptibility gene cluster for ASD.

**Table 1:** Gene Sets Overlapping with the Detected Gene Cluster at a Proportion of ≥50%

Gene Set Name	Overlap Proportion	Gene Set Size
BIOCARTA_PROTEASOME_PATHWAY	0.63	19
REACTOME_SCF_BETA_TRCP_MEDIATED_DEGRADATION_OF_EMI1	0.58	48
REACTOME_P53_INDEPENDENT_DNA_DAMAGE_RESPONSE	0.58	43
NGO_MALIGNANT_GLIOMA_1P_LOH	0.57	7
REACTOME_CYCLIN_E_ASSOCIATED_EVENTS_DURING_G1_S_TRANSITION	0.57	58
REACTOME_SIGNALING_BY_WNT	0.57	58
REACTOME_CYTOSOLIC_TRNA_AMINOACYLATION	0.57	23
REACTOME_SCF_SKP2_MEDIATED_DEGRADATION_OF_P27_P21	0.56	52
REACTOME_VIF_MEDIATED_DEGRADATION_OF_APOBEC3G	0.55	47
BIOCARTA_SRCRPTP_PATHWAY	0.55	11
BIOCARTA_SET_PATHWAY	0.55	11
REACTOME_STABILIZATION_OF_P53	0.54	46
IIZUKA_LIVER_CANCER_PROGRESSION_L0_L1_DN	0.54	13
REACTOME_REGULATION_OF_ORNITHINE_DECARBOXYLASE	0.53	47
KEGG_PROTEASOME	0.52	48
REACTOME_ORC1_REMOVAL_FROM_CHROMATIN	0.51	63
GILMORE_CORE_NFKB_PATHWAY	0.50	10
FIRESTEIN_CTNNB1_PATHWAY_AND_PROLIFERATION	0.50	8
KUROKAWA_LIVER_CANCER_EARLY_RECURRENCE_DN	0.50	6
BIOCARTA_CDMAC_PATHWAY	0.50	16
REACTOME_SHC_MEDIATED_SIGNALLING	0.50	12

This table lists the gene sets with an overlapping proportion ≥50% for the gene cluster detected by the proposed test. For this analysis, we used all 3272 curated gene sets in MsigDB (ver 3.0) [24].

Finally, we compared our proposed method with Fisher's exact test on a 2 × 2 table with rows corresponding to the inside and outside of a predefined gene set, and columns corresponding to cases and controls (Table three b). These are not strict comparisons, because the nature of these hypotheses are different. Our proposed method pertains to the null hypothesis of no gene cluster being associated with a disease in a gene pathway, versus the Fisher's exact test, which pertains to the null hypothesis of no association between a disease and a predefined gene set. However, the general goal is to select gene sets with biological relevance. Results of the Fisher's exact test for deletions are given in Table 2 where we used the Holm's method and the Benjamini-Hochberg false discovery rate (FDR) method to correct for testing all 3272 gene sets. Table 2 illustrates that all nominal associations disappeared after adjusting multiple testing by the Holm's method, with all tests having p-values of more than 0.05. Using a relatively relaxed threshold of 0.2 FDR (q-value), the top seven gene sets appeared significantly associated with ASD. These seven gene sets are not among the gene sets overlapping with the detected gene cluster (Table 1). It should be noted that the gene sets in Table 1 were selected based on an overlap with the detected gene cluster, while the gene sets in Table 2 were selected based on an association with ASD. Therefore, to compare these two methods, a direct comparison between the detected gene cluster and the gene sets in Table 2 would be preferable. For this comparison, Table 2 also illustrates some overlap, though a relatively small one, with the detected gene cluster. This is presumably because these gene sets are not strongly associated with ASD.

**Table 2:** The 20 Most Significant Gene Sets from the ASD Dataset for Deletions

Gene Set Name	nominal *p-value*	FDR *q-value*	Holm's *p-value*	Overlap proportion
NIKOLSKY_BREAST_CANCER_8Q23_Q24_AMPLICON	1.876E-05	0.061	0.061	0.051
PEREZ_TP53_TARGETS	9.768E-05	0.078	0.319	0.019
REACTOME_RNA_POLYMERASE_I_III_AND_MITOCHONDRIAL_TRANSCRIPTION	1.026E-04	0.078	0.336	0.092
ONKEN_UVEAL_MELANOMA_UP	1.065E-04	0.078	0.348	0.098
BROWNE_HCMV_INFECTION_24HR_DN	1.187E-04	0.078	0.388	0.065
REACTOME_RNA_POLYMERASE_I_PROMOTER_CLEARANCE	1.490E-04	0.081	0.487	0.098
BLALOCK_ALZHEIMERS_DISEASE_INCIPIENT_UP	1.937E-04	0.091	0.633	0.052
STARK_HYPPOCAMPUS_22Q11_DELETION_DN	0.001	0.218	1.000	0.115
KEGG_GAP_JUNCTION	0.001	0.218	1.000	0.133
DAIRKEE_TERT_TARGETS_UP	0.001	0.218	1.000	0.111
BLALOCK_ALZHEIMERS_DISEASE_UP	0.001	0.229	1.000	0.062
RODWELL_AGING_KIDNEY_UP	0.001	0.229	1.000	0.067
WALLACE_PROSTATE_CANCER_RACE_UP	0.001	0.268	1.000	0.035
AMUNDSON_RESPONSE_TO_ARSENITE	0.001	0.284	1.000	0.105
NIKOLSKY_BREAST_CANCER_16P13_AMPLICON	0.001	0.284	1.000	0.050
CHARAFE_BREAST_CANCER_LUMINAL_VS_MESENCHYMAL_UP	0.001	0.284	1.000	0.039
FLECHNER_BIOPSY_KIDNEY_TRANSPLANT_REJECTED_VS_OK_DN	0.001	0.284	1.000	0.123
IZADPANAH_STEM_CELL_ADIPOSE_VS_BONE_UP	0.002	0.327	1.000	0.045
BUYTAERT_PHOTODYNAMIC_THERAPY_STRESS_UP	0.002	0.341	1.000	0.057
HSIAO_HOUSEKEEPING_GENES	0.002	0.360	1.000	0.260

This table lists the 20 most statistically significant gene sets as determined by a one-tailed Fisher's exact test, according to Pinto [12]. This analysis was based on the 2 × 2 contingency table shown in Table 3b. Multiple testing was adjusted using Benjamini-Hochberg and Holm's procedures. Results of unadjusted and adjusted tests (Benjamini-Hochberg and Holm's procedure) are referred to as nominal *p-value*, FDR *q-value*, and Holm's *p-value*, respectively. Overlap proportions with the gene cluster detected by the proposed test are referred to as overlap proportion.

### Type I error rate and power

**Figure 2:**
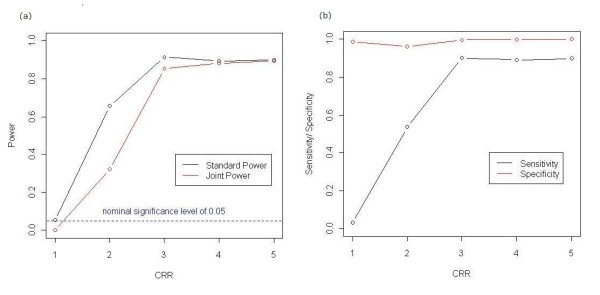
**Empirical Type I Error Rate, Power, Sensitivity, and Specificity of the Proposed Test**. We displayed the type I error rate and power for the proposed test at the nominal significance level of 5% as a function of CRR. The estimation was performed at the nominal significance level of α = 0.05 over 1000 independent simulation datasets of 1000 cases and 1000 controls. (a) Solid black and red lines denote the standard and joint power, respectively, at CRR = 2.0, 3.0, 4.0, or 5.0. The standard power at CRR = 1.0 represents the type I error rate. The dashed blue line indicates the nominal significance level of 0.05. (b) Solid black and red lines show sensitivity and specificity at each CRR, respectively.

Subsequently, we conducted a simulation study to assess the type I error rate and power of the proposed approach. First, we estimated the standard statistical power, which is the probability that the null hypothesis is rejected at a significance level of α = 0.05, without considering the overlap between the detected and real clusters. Permutations of case-control status were used to obtain the critical values of scan statistics. For α = 0.05, this value is defined as the 50th highest scan statistic when 999 permutated replicates are used. The estimated power then equals the proportion of 1000 simulated datasets that have a scan statistic higher than the critical value obtained from the permutated replicates. For the simulated datasets generated at a cluster risk ratio (CRR) of 1.0, the proportion defined above is, in turn, the type I error rate (Figure 2). An empirical type I error rate of 0.054, which is close to the nominal level, was achieved.

To evaluate the performance of the scan statistic for cluster detection, the standard power was derived in the same manner as that for usual hypothesis tests. However, it should be noted that the standard statistical power reflects the "power to reject the null hypothesis for whatever reasons," while the probability of both rejecting the null hypothesis and accurately identifying the true gene cluster is a different matter altogether. Therefore, for this purpose, we used the *joint power*, which can be defined as the probability of accurately detecting the true cluster under an alternative hypothesis [28,29].

The joint power is more suitable for evaluating the accuracy of scan statistics for detecting clusters than the standard power, but the joint power is too stringent to measure the accuracy of cluster detection. For example, even if the gene cluster detected by this test is slightly larger (or smaller) than the true gene cluster, the gene cluster detected is considered as "failure to detect" in terms of the joint power, although the accuracy of cluster detection is considerably high in reality. Therefore, as additional measures of the accuracy of the test, we considered sensitivity and specificity [29]. First, we defined the sensitivity of cluster detection tests as the probability of detecting genes that actually constitute the true gene cluster, *i.e.*, the average proportion of the number of genes in the true gene cluster that are included in the detected gene cluster. Similarly, we defined the specificity of cluster detection tests as the probability of not detecting the genes that do not actually constitute the gene cluster, *i.e.*, the average proportion of the number of genes outside the true gene cluster that are not included in the detected gene cluster.

Both summary measures are better the larger they are, with 1.0 being the optimal. Figure 2 shows all these measures of the test for simulation datasets generated under each CRR. The standard power and sensitivity of the proposed test eventually plateaued around 0.9 (0.914 and 0.901, respectively) at CRR = 3.0. However, even if CRR exceeds 3.0, the joint power tends to increase slightly with increasing CRR, and ends up at the same level of 0.894 at CRR = 5.0. In contrast, the levels of specificity are quite high across all CRRs (>0.95) because the number of genes outside the true gene cluster (13,681 genes) is much higher than those falsely detected (typically, dozens of genes).

Finally, we compared the expected number of genes affected by CNVs (gene count) per case and control in the "true" gene cluster with each observed number. For calculating the expectations, we used the expectation for the binomial distribution, as shown in the Appendix section. Based on a mutation rate of *p = *1.0 × 10^-4^, cluster size M = 100-776, prevalence P(D = 1) = 1%, and CRR γ = 1.0-5.0, the expected number of gene counts per case and control in the "true" gene cluster is comparable to each observation per case and control, respectively, although a smaller cluster size is preferable to fit the observations (Table four).

## Discussion

Using a scan statistic framework, we proposed a novel method to extract gene clusters associated with a specific disease from a global gene pathway. Unlike conventional methods of gene set analyses, our method completely considers the topology of the gene pathway. In the global gene pathway (known as the *interactome*), each gene has a specific role determined by its position. Gene set analyses dismiss such topological pathway information, which generally reduces statistical power. In fact, when we tested whether each gene set was more frequently affected by deletions in ASD cases compared with the controls using Fisher's exact test, statistical significance disappeared after adjustment for multiple testing, which indicated a severe loss of power (Table 2). In contrast, our method detected the gene cluster showing a statistical significance for deletions, although the result was not adjusted for testing twice (both for all CNVs and deletions, *P = *0.025).

Another problem with gene set analysis is overlap between gene sets. Typically, many gene sets share the same genes. For example, the gene sets listed in Table 1 considerably overlap, as shown in Figure 1. This overlap makes interpretation of results from gene set analyses difficult. However, this problem can be completely avoided by extracting one or a few gene clusters from the whole gene pathway. In contrast to approaches using a predefined gene set, it is difficult for this approach to yield meaningful biological interpretations of identified gene clusters.

To interpret the detected gene clusters from a biological point of view, we examined overlap (at least 50%) with predefined gene sets (Table 1). These overlapping gene sets can then be used to interpret detected gene clusters using the consideration of each overlapped proportion as a type of "weight". Although we believe this interpretational method is sufficient, it is also possible to identify overlapping gene sets more formally, using Fisher's exact test on the cross-tabulation of the number of affected genes (gene counts) in/out of a detected gene cluster and by gene counts in/out of a gene set.

In general, scan statistics are used for the scanning of time and space to look for clusters of events (*e.g.*, disease occurrence). The idea is to scan a small window over the whole "map" and to calculate some locality statistic for each window. The maximum of these locality statistics is then defined as the scan statistic.

In this study, we chose the test statistic for testing the difference between two proportions as a locality statistic for calculation ease; nevertheless, other locality statistics can be considered. For example, a locality statistic might be the *p-value *derived from a one-tailed Fisher's exact test in this study setting. Moreover, for each locality statistic, we can use a 2 × 2 table with columns that correspond to cases and controls, and rows that correspond to the number of subjects with or without at least one CNV in each window (Table 3a). This approach has been previously proposed for the analysis of rare single nucleotide variants [16], but it is less powerful than a method based on the number of rare variants (Table 3b) in the context of a quantitative trait [18]. Therefore, we employed the latter "gene count" approach. The locality statistic used in the present study is the same as the cumulative minor-allele test (CMAT) statistic, which was developed for analyzing rare nucleotide variants [19]. Note that other statistics that collapse genotypes at multiple rare nucleotide variants into a univariate test may be used as a locality statistic for the scan statistic proposed here [20,21].

**Table 3:** Contingency Table of Rare CNVs in a Window for a Case-Control Sample

(a)		
	**Cases**	**Controls**

Subjects with at least One Affected Gene in *Z*	*ΣI(x*_*i*_*(Z))*	*ΣI(y*_*i*_*(Z))*
Subjects without any Affected Genes in *Z*	*m*_*1 *_- *ΣI(x*_*i*_*(Z))*	*m*_*0 *_- *ΣI(y*_*i*_*(Z))*
All Subjects	*m*_*1*_	*m*_*0*_

(b)		

	Cases	Controls

Affected Genes in *Z*	*Σx*_*i*_*(Z)*	*Σy*_*i*_*(Z)*
Affected Genes outside *Z*	*n*_*1 *_- *Σx*_*i*_*(Z)*	*n*_*0 *_- *Σy*_*i*_*(Z)*
All Affected Genes	*n*_*1*_	*n*_*0*_

(a) This table summarizes the cross-tabulation between the disease status (cases vs. controls) and the location of each gene affected by CNVs (inside vs. outside a scanning window *Z*) in terms of subject number. *x*_*i*_*(Z) *and *y*_*i*_*(Z) *denote to the number of genes in a window *Z *that were affected by CNVs of the *i*th case and control, respectively. *m*_*1 *_and *m*_*0 *_denote the total number of subjects with genes affected by CNVs in cases and controls, respectively. "Affected genes" mean genes affected by CNVs in a sample. *I(a) *is an indicator variable taking the value 1 if *a >*0 and 0 otherwise.

(b) This table summarizes the cross-tabulation between disease status (cases vs. controls) and the location of each gene affected by CNVs (inside vs. outside a scanning window *Z*) in terms of a gene count. *x*_*i*_*(Z) *and *y*_*i*_*(Z) *denote the number of genes in a window *Z *that were affected by CNVs of the *i*th case and control, respectively. *n*_*1 *_and *n*_*0 *_denote the total number of genes affected by CNVs in cases and controls, respectively. "Affected genes" mean genes affected by CNVs.

Although scan statistics make no assumptions about the shape of the scanning window in general, we used a circular window for computational feasibility. However, tests using circular scan statistics make it difficult to correctly detect non-circular clusters, and tend to detect a larger cluster than the true one by absorbing surrounding regions (genes) where there is no elevated risk [28], which leads to a decrease in power, sensitivity, and specificity. In fact, the gene cluster detected in the ASD dataset is relatively large but may, in fact, be smaller than estimated. Therefore, scan statistics using flexibly shaped windows are preferred, although a more efficient algorithm is needed for practical feasibility.

Next, to evaluate the performance of the proposed scan statistic, we conducted a simulation study based on a simplified scenario, which assumes that genes causative for a disease will be localized proximally to each other in a gene pathway, resulting in one causal gene cluster [25]. This simulation also assumes that any one variant in these genes may lead to disease [19]. In this scenario, the simulation revealed that the statistic shows a high level of accuracy in detecting circular clusters with CRR ≥ 3.0, where CRR is defined as the risk of developing a disease in a person with at least one CNV in a causal gene cluster versus a person with no CNV in that cluster.

Based on the simulation scenarios used here, the expected number of genes affected by CNVs per case and control in a "true" gene cluster is comparable to each observation per case and control, respectively, although a smaller cluster size is preferable to fit the observations (Table 4). Thus, the simulation scheme used appears reasonable to some extent, although other radii, sizes, and locations of gene clusters were not investigated.

**Table 4:** Expected Number of Genes Affected by Deletions in the Most Likely Gene Cluster per Case and Control Compared with Each Observation


(1) Cluster size = 776						

	Observations			CRR		
		1.0	2.0	3.0	4.0	5.0

Case	0.054	0.078	0.144	0.203	0.203	0.254
Control	0.011	0.078	0.077	0.076	0.076	0.076

(2) Cluster size = 500						

	Observations			CRR		
		1.0	2.0	3.0	4.0	5.0

Case	0.054	0.050	0.095	0.137	0.174	0.209
Control	0.011	0.050	0.050	0.049	0.049	0.048

(3) Cluster size = 300						

	Observations			CRR		
		1.0	2.0	3.0	4.0	5.0

Case	0.054	0.030	0.058	0.085	0.110	0.134
Control	0.011	0.030	0.030	0.029	0.029	0.029

(4) Cluster size = 200						

	Observations			CRR		
		1.0	2.0	3.0	4.0	5.0

Case	0.054	0.020	0.039	0.058	0.076	0.093
Control	0.011	0.020	0.020	0.020	0.019	0.019

(5) Cluster size = 100						

	Observations			CRR		
		1.0	2.0	3.0	4.0	5.0

Case	0.054	0.010	0.020	0.029	0.039	0.048
Control	0.011	0.010	0.010	0.010	0.010	0.010

This table compares the expected number of genes affected by CNVs in the most likely gene cluster per case and control with each observation. The expected numbers were calculated from the disease model for CRR = 1.0, 2.0, 3.0, 4.0, or 5.0 and cluster size = 100, 200, 300, 500, or 776 (776 = size of the most likely cluster).

With regard to a gene pathway, in this study, we treated a gene pathway as an undirected graph, where the direction of the interaction (edge) between genes (nodes) is not specified. In addition, we defined the shortest paths among all pairs of nodes as distances, which were calculated using the Dijksta's algorithm implemented in the RBGL package [34]. This definition of distance is commonly used in the functional genomics field [35], but other definitions should also be investigated in terms of graph theory.

Finally, as our method is expected to strongly depend on the spatial configuration of genes, an extensive and reliable functional gene pathway is crucial for its performance. Our knowledge of human pathways is still far from complete, and thus, the extent to which our method is influenced by incomplete information remains to be investigated.

## Conclusions

This study has provided a sound statistical framework for analyzing genome-wide rare CNV data by incorporating gene pathway information. The scan statistic approach proposed here shows a high level of accuracy in detecting gene clusters in a whole gene pathway. For other settings, this framework can be easily applied by choosing locality statistics suitable for the desired purposes. This proposed method is not restricted to CNVs and can, in principle, be used for analyzing genome-wide resequencing data. With the amount and quality of gene pathway information expanding rapidly, the method of handling such information in a statistically proper manner is becoming increasingly important for analyzing rare variants, including rare CNVs.

## Methods

### Scan statistics and permutation tests

Let us consider the gene pathway defined by the Pathway Commons metadatabase, which integrates nine publicly available biological pathways [30]. This pathway of *Homo sapiens *contains 13,682 genes (or their encoded proteins) and 538,610 protein-protein interactions in all (September 7, 2010), and we use this pathway for our analyses and simulations. This pathway is represented by a set of nodes and set of edges between these nodes. The nodes represent gene products, *e.g.*, individual proteins. There is an edge from node A to node B if A transfers the signal it received immediately to B in the case of a signaling pathway (*e.g.*, changing the phosphorylation state of B) or if A and B catalyze two successive reactions (*e.g.*, metabolic pathways). To take into account the internal structure of the pathway and to give rigorous meaning to the "closeness" between genes, we defined the distance between nodes (genes) as follows: assume that each edge represents a unit distance between two nodes and that the shortest way to connect two nodes via two edges in the pathway implies two units of distance. If more than one path connects two nodes, the shortest distance between the two nodes indicates the distance between them. Note the phrase "distance between a pair of nodes" is used to imply the "shortest distance between this pair".

**Figure 3:**
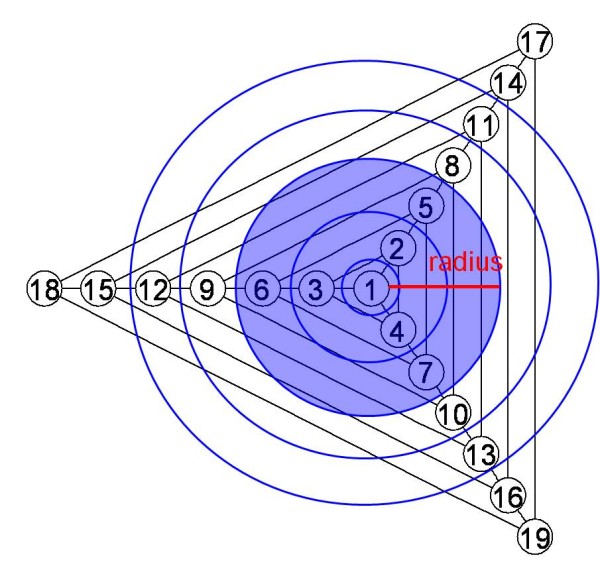
**A Diagram Showing Scan Windows**. All the nodes with numbers 1-19 are genes, and the edges represent any kind of interaction between two genes. Circles centered on node 1 represent scan windows to search over, with a continuously varying radius from zero to the preset upper limit *R *= 4. For example, a blue circle indicates a window centered on node 1 with a radius of two.

**Figure 4:**
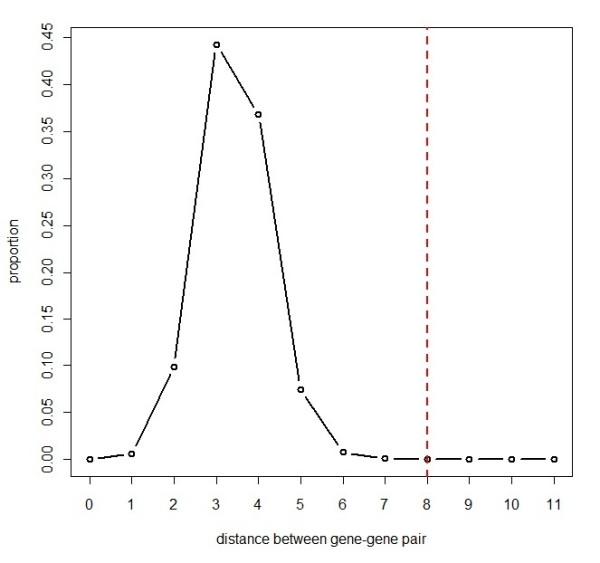
**Distribution of Distances between All Gene Pairs**. The solid line indicates the distribution of distances between all possible gene pairs in the gene pathway, which was defined by Pathway Commons (*Homo sapiens*) [30]. The vertical dashed line denotes the preset maximum diameter (= 2 × the maximum radius) of windows. Considering all windows of maximum diameter, more than 99.9% of genes in the gene pathway analyzed were covered by at least one of the windows.

To detect gene clusters associated with a disease in the pathway, we can choose a set of windows to search over, where each window consists of a set of one or more genes. Note that throughout this paper we use the term "gene set" as a set of genes with a common biological functionality, exemplified by Table 1 and Table 2. By contrast, the term "window" only refers to a set of genes to test and does not necessarily possess such a clear functionality. In principle, the number of windows searched can be as high as 2^*N*^, where *N *is the number of all genes in the pathway (*i.e.*, 13,682). However, such a huge number of windows is not computationally feasible. Therefore, in practice, we enforced constraints on the size and shape of windows to make their number much smaller. In this study, we considered circular windows centered on each gene with a continuously varying radius from zero to a possible upper limit *R *(Figure 3). This constraint markedly reduces the number of windows to (*R + *1*)N*. Because the windows of a maximum radius *R *= 4 include more than 99.9% of all genes in the pathway (Figure 4), we set *R = *4 in the present study. This drastically decreases the number of windows from virtually infinite to just about 68,000. We define a set of windows ***Z***, where each window *Z *∈ ***Z ***is set as circular on each gene, and the radius of the windows vary from zero to the preset maximum *R *= 4 (Figure 3, see the Appendix section).

Our method compares the proportion of genes affected by rare CNVs in each window between cases and controls. If a window (gene cluster) is associated with a disease, the proportion of affected genes in this window substantially differs between cases and controls. Let φ_case_(*Z*) and φ_control_(*Z*) denote the proportion of genes affected by rare CNVs in cases and controls, respectively, which are contained in a window *Z*. The standard test statistic for testing the difference between two proportions can then be used to test the null hypothesis: φ_case_(*Z*) = φ_control_(*Z*).

Merely testing the null hypothesis would provide us a large number of windows to test, which would create the problem of multiple testing. To avoid this problem, we set the null hypothesis H_0 _of no gene cluster associated with the disease in the gene pathway, and we set the alternative hypothesis stating that there is at least one window *Z *for which the proportion of affected genes is higher in cases than in controls.

Here we employed a one-tailed test because it appeared unlikely that rare CNVs have protective effects against diseases [14].

Let *n*_*1 *_denote the total number of genes affected by rare CNVs in cases and let x_i_(Z) denote the number of genes in a window Z affected by rare CNVs of the *i*th case. Similarly, let *n*_*0 *_denote the total number of genes affected by rare CNVs in controls and let y_i_(Z) denote the number of affected genes in a window *Z *of the *i*th control. In the window *Z*, the number of affected genes carried by each phenotypic group is Σ_i_x_i_(Z) and Σ_i_y_i_(Z). The data are then summarized in a 2 × 2 contingency table (Table 3b), and the scan test statistic for the above hypothesis is simply given by the maximum locality statistic for a window Z:

Where , and . Note that we calculated the statistic only for windows of ∑_*i*_*x*_*i *_(*Z*)>0 to avoid a denominator = 0.

To determine the distribution of the test statistic under the null hypothesis, we performed a permutation test. In this study, the p-value of the test is determined on the basis of the null distribution of the test statistic with a large number (we used 999) of permutation replicates, where the labels "case" and "control" are permuted.

If the test of the single most significant cluster (hereafter referred to as the "most likely cluster") is statistically significant, there is interest in testing for the presence of additional clusters ("secondary clusters"). To derive secondary clusters in addition to the most likely cluster, locality statistics for each window Z are arranged in descending order. The first (largest) locality statistic is a scan statistic for the most likely cluster, and the second (largest) locality statistic is a scan statistic for the second most likely cluster and so on. However, because expanding or reducing the cluster size will only marginally alter the locality statistic, there will almost always be a second most likely cluster that is almost identical to the most likely cluster. Most clusters of this type provide little additional information. Thus, the most interesting secondary clusters are the windows Z that do not overlap with the most likely cluster and that are statistically significant. To test for secondary clusters that do not overlap with the most likely clusters, p-values for secondary clusters are calculated using the null distribution of the scan statistic for the most likely clusters. Because the locality statistic for the secondary cluster is less than that of the most likely cluster, the p-values for secondary clusters are typically conservative to some extent [27]. This procedure is then repeated until there are no more clusters with p-values less than a significance level, α = 0.05.

### Simulation scheme

First, we describe a novel way of simulating case-control genome-wide rare CNV data. We assume that a rare variant resides on one gene (*i.e*., no rare variant resides on more than two genes) and that *M *genes are included in a single causal gene cluster. Then, we denote the genotype at the *j*th gene in the causal gene cluster by g_j_, and the joint genotypes for M disease genes in the cluster by ***g ****= {g*_*1*_*,g*_*2*_*,...,g*_*M*_*}*. Because a homozygote for a minor allele is extremely rare at each variant site because of its rarity, the genotype for an individual at the *j*th disease variant is denoted by *g*_*j*_*{*0,1*}*, where 0 denotes the wild-type genotype (homozygous for a major allele) and 1 denotes a heterozygote. We use *D *= 1 to denote a case individual and *D *= 0 to denote a control. According to Wright [36], let a referent genotype ***g***_**0 **_denote the joint genotype = 0 at all sites. If we specify the population genotype frequencies *P(****g****)*, the disease prevalence *P(D *= 1*)*, and the risk ratio *RR(****g****) = P(D *= 1*|****g****)/(D *= 1*|****g***_***0***_*) *for all ***g***, then we get the following equation:

For the disease model, we assume that a rare mutation that disrupts any one of *M *genes in the causal gene cluster may well lead to common diseases; therefore, we adopted RR(***g***) = constant as the risk model in our simulation; say, γ for ***g ****≠ ****g***_***0 ***_and *RR(****g****) = *1 for ***g ***= ***g***_***0***_. Thus, for ***g ***≠ ***g***_***0***_

Hereafter, we refer to γ as the cluster risk ratio (CRR). Furthermore, we have

in which all terms on the right-hand side are known.

In the disease model, we assume that in the source population, all variant sites in the considered gene cluster are independent. We also assume that genotypes = 1 at each variant site are generated with a nearly constant probability of *p*. This assumption is not required for our method but is used only for the ease of simulation. Let ***g***_***i ***_refer to a joint genotype with *i *genotypes of 1 and *(M-i) *genotypes of 0. We can then approximate the population genotypic frequencies by the binomial distribution as follows:

Therefore, for ***g***_***i ***_≠ ***g***_***0***_,

Thus, we can simulate the joint genotype of a causal gene cluster for cases and controls by the following two steps: (1) calculate *P(****g***_***i***_*|D =*1*) *and *P(****g***_***i***_*|D =*0*) *for all ***g***_***i ***_and (2) randomly select *i *mutated genes from a causal gene cluster with the probability of *P(****g***_***i***_*|D *= 1*) *for a case subject and with the probability of *P(****g***_***i***_*|D *= 0*) *for a control subject.

For genes not included in a causal gene cluster, it follows from this model that *P(****g***_***i***_*|D *= 1*) = P(****g***_***i***_*|D *= 0*) = P(****g***_***i***_*)*, and the joint genotypes outside a causal gene cluster can be obtained according to the above steps. Finally, by combining joint genotypes inside and outside a causal gene cluster, we can create entire joint genotypes for both cases and controls.

In all our simulations, we generated 1000 datasets with each consisting of 1000 cases and 1000 controls. Based on previous studies [37,38], the CNV mutation rate, *p*, is set as 1.0 × 10^-4 ^because our study focuses only on rare CNVs with relatively homogeneous frequency spectra. To simulate ASD, we set the disease prevalence to 1.0% [39,40]. We considered CRR = 1.0, 2.0, 3.0, 4.0, or 5.0 in reference to several reported CNVs with quite high odds ratios of 2.7-21.6 [3,4]. For the true causal gene cluster, we chose the gene cluster detected in the ASD dataset analyzed above that contains 776 genes within the radius of two (Additional file 1), *i.e.*, M = 766 in the simulation.

The program for computation of the scan statistic was developed for this work and implemented in C on Windows XP. This program is available on request to the authors. All the other computations and simulations were conducted on the same computer, using R programming language [41] ver 2.9.0.

## Abbreviations

CNV: copy number variant (variation); ASD: autism spectrum disorder; MSigDB: molecular signatures database; CRR: cluster risk ratio.

## Authors' contributions

TN, TK, TT, and HK conceptualized the study, designed the statistical model, performed the data analyses, and wrote the manuscript. ST developed the software. DP and SWS provided the case-control data and supervised their analyses. All authors read and approved the final manuscript.

## Appendix

### The procedure for choosing a set of genes (window) Z to search over

1. We first obtain a distance matrix d_kl_, which is defined by shortest distance between gene k and gene l (*k, l = *1*,..., N*). By focusing on the *k*th gene (row), we choose a set of genes (window) Z with distances equal to or smaller than the radius r (*i.e*., d_kl _≤ r). Then, we compute a scan statistic T(Z) for the chosen window.

2. Next, by increasing the radius r to the predefined upper limit R, we find the window with the highest value of T(Z) that is centered on the *k*th gene.

3. Finally, by moving the *k*th centered gene (row) from 1 to N, we find the set of genes with the globally highest value of T(Z).

### The expected number of genes affected by CNVs (gene count) per case and control

Here we provide the equation for the expected gene count in a disease gene cluster per case and control. Following the argument of method section, we have

where *K *equals disease prevalence, *P(D *= 1*)*.

Therefore, the expected gene count in a case individual is given by:

Similarly, the expected gene count in a control is given by:

## Supplementary Material

Additional file 1 **Additional file 1.doc**. The Gene Cluster Detected in the Whole Gene Pathway by the Proposed Test Click here for file

Additional file 2 **Additional file 2.doc**. Genes Detected by the Proposed Test for Deletion Click here for file
