# Supplementary material for: A scan statistic to extract causal gene clusters from case-control genome-wide rare CNV data
Source: BMC Bioinformatics. 2011 May 26;12:205. doi: 10.1186/1471-2105-12-205 (PMC3130692; doi:10.1186/1471-2105-12-205)

**Additional file1.**

**Figure S1. The Gene Cluster Detected in the Whole Gene Pathway by the Proposed Test**

Blue and light blue nodes represent genes inside and outside the gene cluster detected by the proposed test for the ASD dataset, respectively [12]. This gene set contains 776 genes centered on the ACAT1 gene, which is represented by a red node. Thin black lines represent edges (interactions) between genes.


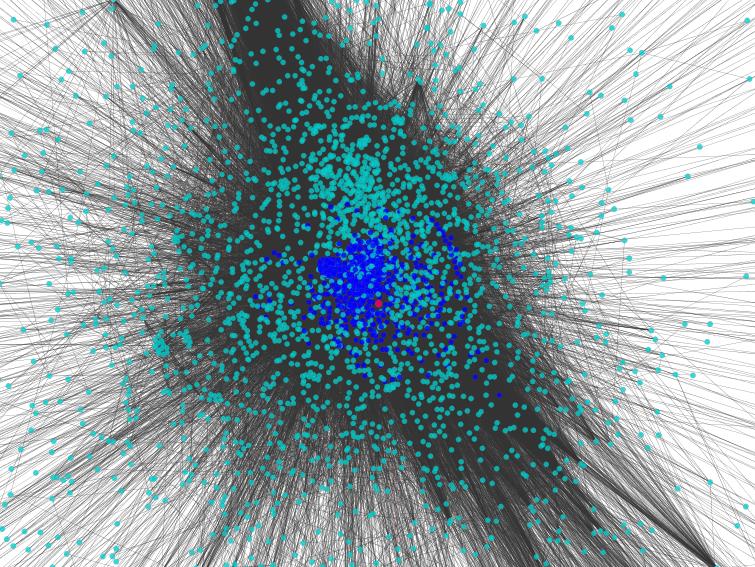

Supplement: Additional file 1 — doc. The Gene Cluster Detected in the Whole Gene Pathway by the Proposed Test [file 1471-2105-12-205-S1.DOC]
