# Supplementary material for: A scan statistic to extract causal gene clusters from case-control genome-wide rare CNV data
Source: BMC Bioinformatics. 2011 May 26;12:205. doi: 10.1186/1471-2105-12-205 (PMC3130692; doi:10.1186/1471-2105-12-205)
Supplement: Additional file 2 — doc. Genes Detected by the Proposed Test for Deletion [file 1471-2105-12-205-S2.DOC]

**Additional file2.**

Table S1. Genes Detected by the Proposed Test for Deletion

| AAK1 | AASDHPPT | ABL2 | ACACA | ACAT1 | ACAT2 | ACOT13 | ACOX3 |
| --- | --- | --- | --- | --- | --- | --- | --- |
| ACP1 | ACTB | ACTN4 | ACTR1A | ACTR2 | ACTR3 | ACVR1 | ACVR1B |
| ACVR2B | ADAM28 | ADAR | ADCK1 | ADCY5 | ADK | ADRM1 | ADSS |
| AFF4 | AFP | AHCY | AHNAK | AHSA1 | AIFM1 | AIMP1 | AIMP2 |
| AK2 | AKAP9 | AKR1B1 | AKR7A2 | ALDH9A1 | ALDOA | ALPK3 | ANAPC5 |
| ANAPC7 | ANP32A | ANXA2 | AP2A1 | AP2B1 | AP2M1 | APEH | APEX1 |
| APRT | ARAF | ARF6 | ARL1 | ARPC2 | ARPC4 | ATAD3B | ATF2 |
| ATF7IP | ATP5A1 | ATP5B | ATP5C1 | ATP5D | ATP5L | ATP5O | ATP6V1A |
| AURKA | AURKB | AXIN2 | AZI1 | AZI2 | B2M | BANF1 | BCAP31 |
| BCAS2 | BMP2K | BMPR1A | BRAF | BRD3 | BTF3 | BTK | BTRC |
| BUB3 | C12orf23 | C1QBP | C4orf43 | CABC1 | CACYBP | CAD | CALM1 |
| CALR | CAMK2B | CAMK2D | CAMK2G | CAMKK2 | CAPRIN1 | CAPZA1 | CAPZB |
| CBR1 | CCNA1 | CCNH | CD3EAP | CD81 | CD8A | CDC37 | CDK1 |
| CDK12 | CDK13 | CDK2 | CDK4 | CDK5 | CDK7 | CDK9 | CDKN2A |
| CHUK | CISD2 | CKMT1A | CLIC1 | CLIC4 | CLK1 | CLPP | CLTC |
| CLU | CMPK1 | CNBP | CNKSR2 | CNPY2 | COMT | COPB2 | COPG |
| COPS3 | COPS6 | COPZ1 | CPS1 | CPSF6 | CRY1 | CRY2 | CRYZ |
| CS | CSDE1 | CSF1 | CSK | CSNK1A1 | CSNK1D | Csnk1e | CSNK1E |
| CSNK1G3 | CSNK2A1 | CSNK2A2 | CSNK2B | CSTB | CTNNB1 | CTPS | CUL1 |
| CUL2 | CUTA | CYB5B | CYCS | DAD1 | DCK | DCTPP1 | DCUN1D1 |
| DDOST | DDT | DDX21 | DDX3Y | DDX5 | DFFA | DHCR7 | DHX36 |
| DHX9 | DIABLO | DISC1 | Dlg4 | DLST | DNAJA1 | DNAJB6 | DNAJC8 |
| DNM2 | DOK1 | DOK2 | DRG1 | DSG1 | DSP | DSTN | DUSP3 |
| DUT | DYNLL1 | DYNLRB1 | DYRK1A | ECH1 | ECHS1 | EEF1B2 | EEF1D |
| EEF1E1 | EIF1AX | EIF1B | EIF2AK1 | EIF2AK4 | EIF2B1 | EIF2S1 | EIF2S3 |
| EIF3A | EIF3D | EIF3E | EIF3F | EIF3G | EIF3H | EIF3I | EIF3J |
| EIF3K | EIF3L | EIF3M | EIF4G1 | EIF4H | EIF6 | EPHB1 | EPHB2 |
| EPHB4 | EPRS | ERH | ERLEC1 | ERP29 | ESD | ETFA | EWSR1 |
| EXOSC4 | EZR | F8 | FABP5 | FAM110A | FAM110B | FAM40A | FAM83A |
| FAM83H | FBLN1 | FBXW11 | FER | FGFR1 | FHL1 | FKBP3 | FRYL |
| FSCN1 | FTSJ1 | FYN | G3BP1 | G3BP2 | GAK | GANAB | GAPDH |
| GAPVD1 | GARS | GCLM | GDI1 | GDI2 | GFPT1 | GH1 | GHR |
| GLOD4 | GLRX3 | GLRX5 | GLUL | GMPS | GNB1 | GNPDA1 | GOLGA4 |
| GOT1 | GOT2 | GPI | GRAMD1A | GRB2 | GRPEL1 | GSK3A | GSK3B |
| GSPT1 | GSTM3 | HADHA | HARS | HBA1 | HCK | HINT1 | HIPK1 |
| HIST1H1E | HIST1H2BC | HLA-A | HLA-B | HLA-C | HMGB2 | HNRNPA0 | HNRNPA1 |
| HNRNPA3 | HNRNPC | HNRNPD | HNRNPF | HNRNPH1 | HNRNPH2 | HNRNPH3 | HNRNPL |
| HNRNPM | HNRPLL | HPRT1 | HRNR | HSP90AB1 | HSPA4L | HSPA5 | HSPE1 |
| HYOU1 | HYPK | IARS | IARS2 | ICT1 | IGF2BP1 | IKBKAP | IKBKB |
| IMPDH2 | INPPL1 | IQCH | JAK1 | JAK2 | JUP | KATNAL2 | KCTD12 |
| KIAA0090 | KIAA0195 | KIAA0528 | KIAA1618 | KIR2DL1 | KIR2DL2 | KIR2DL3 | KIR2DS1 |
| KIR2DS2 | KIR3DL1 | KIT | KPNA3 | KRT18 | LAGE3 | LARP1 | LETM1 |
| LILRB1 | LILRB2 | LIMK1 | LIMK2 | LIN28A | LMNB1 | LRPPRC | LRRC40 |
| LRRC59 | LSM14A | LTA4H | LTV1 | LYAR | LYN | MACF1 | MAGEA1 |
| MAGOH | MAP2K1 | MAP2K2 | MAP3K11 | MAP3K2 | MAP3K3 | MAP3K4 | MAP4K2 |
| MAP4K5 | MAPK1 | MAPK14 | MAPK3 | MAPK6 | MAPRE1 | MARK1 | MARK2 |
| MARK3 | MARK4 | MARS | MCC | MCM3 | MCM4 | MCM5 | MCM6 |
| MCM7 | MCTS1 | MELK | MFAP2 | MIF | MLEC | MLLT1 | MMS19 |
| MNAT1 | MOBKL1B | MOBKL3 | MPG | MRPL14 | MRPL40 | MRPS16 | MSH2 |
| MSH6 | MSI2 | MSN | MT-CO2 | MTDH | MTHFD1 | MTHFD2 | MTPN |
| MXRA5 | MYC | MYH10 | MYL12A | MYL6 | MYT1L | NAA10 | NAA15 |
| NAA50 | NACAP1 | NANS | NAP1L1 | NASP | NCK1 | NCK2 | NCOR2 |
| NDUFS1 | NEFM | NEK11 | NEK9 | NFKB1 | NFKBIB | NLK | NME1 |
| NME2P1 | NPEPPS | NPM1 | NQO2 | NTRK1 | NUDC | NUDCD2 | NUDT21 |
| NUDT5 | NUP155 | NUP43 | NUTF2 | OAT | OTUB1 | PA2G4 | PABPC1 |
| PAFAH1B2 | PAICS | PAK4 | PARD3 | PCBP1 | PCBP3 | PCMT1 | PCNA |
| PDCD10 | PDCD2 | PDCD5 | PDCD6IP | PDE4A | PDE4DIP | PDHA1 | PDHB |
| PDIA3 | PDIA4 | PDLIM1 | PDPK1 | PEBP1 | PER1 | PER2 | PFAS |
| PFDN2 | PFDN4 | PFDN5 | PFKL | PFKM | PFKP | PFN1 | PFN2 |
| PGD | PGK1 | PGRMC1 | PHB2 | PIN1 | PIN4 | PINX1 | PIP4K2C |
| PKM2 | PKMYT1 | PKN1 | PKN3 | PLK4 | PLS3 | PNP | PNPT1 |
| POLR1B | POLR1D | POLR1E | POLR2H | POLR2L | PPIA | PPIB | PPIH |
| PPP1R13B | PPP2CB | PPP2R1A | PREP | PRKAA1 | PRKAB1 | PRKAB2 | PRKCA |
| PRKCB | PRKCD | PRKCQ | PRKD2 | PRKDC | PRLR | PRMT1 | PRNP |
| PRPSAP1 | PSAT1 | PSMA1 | PSMA2 | PSMA3 | PSMA6 | PSMA7 | PSMB1 |
| PSMB3 | PSMB4 | PSMB6 | PSMB7 | PSMC1 | PSMC2 | PSMC3 | PSMC6 |
| PSMD1 | PSMD12 | PSMD13 | PSMD14 | PSMD2 | PSMD3 | PSMD4 | PSMD6 |
| PSMD7 | PSMD8 | PSME3 | PTGES3 | PTK2 | PTK2B | PTPN18 | PUS7 |
| PYCR2 | RAB14 | RAB1B | RAB21 | RAB2A | RAB5C | RAB6A | RAB7A |
| RAN | RANBP1 | RANGAP1 | RAP1B | RAP2A | RARS | RASAL2 | RBBP4 |
| RBM14 | RBMX | RCC2 | REL | RELA | REXO2 | RHEB | RHOA |
| RHOB | RHOC | RHPN2 | RIPK2 | RNASEH1 | RNMTL1 | RPA1 | RPA2 |
| RPA3 | RPL18A | RPL22 | RPL23A | RPL24 | RPL26 | RPL29 | RPL3 |
| RPL31 | RPL32 | RPL35 | RPL35A | RPL36 | RPL37A | RPL38 | RPLP0 |
| RPN1 | RPN2 | RPS10 | RPS11 | RPS14 | RPS16 | RPS19 | RPS26 |
| RPS28 | RPS29 | RPS3 | RPS3A | RPS6KA2 | RPS6KA3 | RPS9 | RRM1 |
| RRP12 | RUVBL1 | RUVBL2 | S100A11 | SARS | SBDS | SCRIB | SDHA |
| SDHB | SEC11A | SEC13 | SEC16A | SEC23A | SEC63 | SEPHS1 | SERBP1 |
| SET | SF3A1 | SF3A2 | SF3B14 | SFPQ | SFRS1 | SFXN1 | SGTA |
| SHC1 | SHMT2 | SIK2 | SKP1 | SLC1A5 | SLC25A1 | SLC9A1 | SLC9A3R1 |
| SLC9A3R2 | SLIRP | SLK | SMAP | SMC2 | SMC3 | SMC4 | SND1 |
| SNRNP200 | SNRPA | SNRPA1 | SNRPB2 | SNX24 | SNX27 | SNX3 | SOD2 |
| SORD | SPEG | SPTBN1 | SRC | SRGAP3 | SRP14 | SRP68 | SRP72 |
| SRRM2 | SSB | SSR1 | SSR4 | SSRP1 | SSSCA1 | STAU1 | STK16 |
| STK24 | STMN1 | STOML2 | STOX2 | STRAP | STRBP | STRN | STRN3 |
| STRN4 | SUPT16H | SYK | SYMPK | SYNCRIP | TAF15 | TAGLN2 | TALDO1 |
| Tanc1 | TANK | TAOK1 | TAOK3 | TAP1 | TAP2 | TARDBP | TARS |
| TBCE | TBK1 | TBKBP1 | TCEB1 | TCF7L2 | TEAD3 | TEC | TESK2 |
| TFAM | TFRC | TGFBR1 | TIMM13 | TIMM16 | TKT | TLN1 | TMCO1 |
| TMX1 | TNFRSF14 | TNIK | TNK1 | TOMM40 | TP53BP1 | TP53RK | TPM2 |
| TPM3 | TPP2 | TPT1 | TRAF2 | TRAP1 | TRIB3 | TRIM2 | TRIM28 |
| TRIP11 | TRMT112 | TSFM | TSN | TSR1 | TTLL12 | TTN | TUFM |
| TXNDC17 | TYK2 | TYROBP | U2AF2 | UBA2 | UBAC1 | UBE2D1 | UBE2D3 |
| UBE2I | UBE2K | UBE2L3 | UBE2M | UBE2V2 | UBXN1 | UCHL1 | UCHL5 |
| UGGT1 | ULK3 | UQCRC2 | USP14 | VAPA | VARS | VCP | VDAC1 |
| VDAC3 | VEPH1 | VHL | VMA21 | VPS13B | VPS35 | WDR48 | WDR5 |
| WEE1 | WWC1 | YARS | YES1 | ZNF346 | 2-Sep | 4-Sep | 15-Sep |

Analysis of the ASD dataset shows that these 776 genes are included in the most likely gene sets.
